# Rare *KCNQ4* variants found in public databases underlie impaired channel activity that may contribute to hearing impairment

**DOI:** 10.1038/s12276-019-0300-9

**Published:** 2019-08-21

**Authors:** Jinsei Jung, Haiyue Lin, Young Ik Koh, Kunhi Ryu, Joon Suk Lee, John Hoon Rim, Hye Ji Choi, Hak Joon Lee, Hye-Youn Kim, Seyoung Yu, Hyunsoo Jin, Ji Hyun Lee, Min Goo Lee, Wan Namkung, Jae Young Choi, Heon Yung Gee

**Affiliations:** 1 Department of Otorhinolaryngology, Brain Korea 21 PLUS Project for Medical Sciences, Yonsei University College of Medicine, Seoul, 03722 Korea; 2 Department of Pharmacology, Brain Korea 21 PLUS Project for Medical Sciences, Yonsei University College of Medicine, Seoul, 03722 Korea; 3 Yonsei University College of Pharmacy, Incheon, 21983 Korea; 4 Yonsei University College of Medicine, Seoul, 03722 Korea; 5 Department of Dermatology, Seoul St. Mary’s Hospital, College of Medicine, The Catholic University of Korea, Seoul, 03722 Korea

**Keywords:** Genetic variation, Mechanisms of disease, Neurophysiology

## Abstract

*KCNQ4* is frequently mutated in autosomal dominant non-syndromic hearing loss (NSHL), a typically late-onset, initially high-frequency loss that progresses over time (DFNA2). Most *KCNQ4* mutations linked to hearing loss are clustered around the pore region of the protein and lead to loss of KCNQ4-mediated potassium currents. To understand the contribution of *KCNQ4* variants to NSHL, we surveyed public databases and found 17 loss-of-function and six missense *KCNQ4* variants affecting amino acids around the pore region. The missense variants have not been reported as pathogenic and are present at a low frequency (minor allele frequency < 0.0005) in the population. We examined the functional impact of these variants, which, interestingly, induced a reduction in potassium channel activity without altering expression or trafficking of the channel protein, being functionally similar to DFNA2-associated *KCNQ4* mutations. Therefore, these variants may be risk factors for late-onset hearing loss, and individuals harboring any one of these variants may develop hearing loss during adulthood. Reduced channel activity could be rescued by KCNQ activators, suggesting the possibility of medical intervention. These findings indicate that *KCNQ4* variants may contribute more to late-onset NSHL than expected, and therefore, genetic screening for this gene is important for the prevention and treatment of NSHL.

## Deafness: Missed mutations raise risk of hearing loss

A gene associated with hereditary hearing loss may play a greater role than previously recognized in age-related auditory impairment. Many cases of autosomal dominant non-syndromic hearing loss (ADNSHL) arise from defects in KCNQ4, a protein that maintains the cellular ionic conditions needed for normal inner ear function. Researchers led by Heon Yung Gee and Jae Young Choi at Yonsei University College of Medicine, Seoul, South Korea, have now uncovered numerous previously overlooked mutations in the gene encoding KCNQ4 that may also contribute to adult-onset hearing loss. Their survey of human genome databases revealed 23 additional sequence variants that can meaningfully impair function of this protein. The effects of some of these mutations can be at least partially corrected with existing chemical compounds, indicating the potential to protect a subset of ADNSHL patients from future deafness.

## Introduction

KCNQ4 (KV7.4), a voltage-gated potassium channel, plays a critical role in the auditory function of the inner ear by regulating K^+^ recycling and homeostasis, together with other K^+^ ion channels (KCNQ1/KCNE1), gap junctions (GJB2, GJB3, and GJB6), and transporters (Na^+^/K^+^/2Cl^−^)^1,2^. The *KCNQ4* gene was first cloned and characterized by Kubisch et al.^3^. They mapped it to the DFNA2 (autosomal dominant non-syndromic hearing loss; ADNSHL) locus, and a dominant-negative mutation was identified in the DFNA2 pedigree. DFNA2 is characterized as a late-onset disease, with high-frequency hearing loss that progresses to all frequencies over time^3,4^.

*KCNQ4* is one of the most commonly mutated genes in ADNSHL^5^. Approximately 30 pathologic mutations in *KCNQ4* have been identified as the cause of DFNA2 (www.deafnessvariationdatabase.org or www.hgmd.cf.ac.uk/ac/index.php). The KCNQ4 channel consists of six transmembrane domains, a pore region, and two intracellular termini^6^. The mutation hotspots in *KCNQ4* associated with DFNA2 are clustered around the pore region^7^. The first described missense mutation (p.G285S)^3^ as well as p.L274S, p.W276S, p.L281S, p.G285C, and p.G296S, which correspond to changes around the pore region of KCNQ4, induced a loss of channel function or decreased membrane expression of the channel protein^4,8–15^.

Age-related hearing loss (ARHL) is the most common sensory deficit among the elderly and progresses slowly, similar to DFNA2. ARHL is a complex disease resulting from an interplay between genetic and environmental factors^16^. However, the contribution of genetic predisposition to ARHL is not clear and, therefore, often underestimated. Several single nucleotide polymorphisms in *KCNQ4* are significantly associated with ARHL^17^, and it is plausible that the hypomorphic mutations in *KCNQ4* might contribute to ARHL^18^. In addition, *KCNQ4* variants are also implicated in noise-induced hearing loss^19^.

In this study, we examined *KCNQ4* variants in public databases and found several missense variants around the pore region of KCNQ4 that are present at a low frequency in the general population and have not been associated with DFNA2. As the pathogenicity or clinical significance of these variants is unknown, we investigated the effects of these variants on KCNQ4 function and found that they significantly decreased K^+^ channel activity. These findings suggest that these KCNQ4 variants may contribute to NSHL or ARHL.

## Materials and methods

### *KCNQ4* variants

*KCNQ4* variants were examined in several databases: HGMD Professional (http://www.hgmd.cf.ac.uk/ac/index.php), ClinVar (https://www.ncbi.nlm.nih.gov/clinvar/), the Deafness Variation Database (http://www.deafnessvariationdatabase.org/), and the Genome Aggregation Database (gnomAD) (http://gnomad.broadinstitute.org/). A whole-genome sequencing dataset of 397 Korean individuals from the National Biobank of Korea of the Centers for Disease Control and Prevention was also used.

### Plasmid construction and site-directed mutagenesis

Complementary DNAs (cDNAs) of human *KCNQ4* were purchased from OriGene Technologies (Rockville, MD, USA) and subcloned into the pENTR-D-TOPO vector (Invitrogen, Carlsbad, CA, USA). Expression vectors were constructed using LR clonase (Invitrogen) following the manufacturer’s instructions, and a Myg- or FLAG-tag was inserted in the N-terminus. *KCNQ4* variant clones were generated by PCR-based site-directed mutagenesis using the Quick Change II XL Site-Directed Mutagenesis Kit (Agilent Technologies, Santa Clara, CA, USA).

### Cell culture and transfection

Human embryonic kidney 293 (HEK 293) and Chinese hamster ovary (CHO) cells were cultured in Dulbecco’s modified essential medium and RPMI 1640 medium, respectively, supplemented with 10% fetal bovine serum and penicillin (50 IU/mL)/streptomycin (50 μg/mL) (Invitrogen). Cells were transfected with wild-type (WT) or mutant *KCNQ4* plasmids using Lipofectamine and PLUS reagent, or Turbofect (Thermo Fisher Scientific, Waltham, MA, USA), according to the manufacturer’s instructions. For electrophysiological experiments, CHO-K1 cells were cotransfected with 0.9 μg of the human KCNQ4 plasmids with 0.1 μg of a green fluorescent protein (GFP) gene-containing expression plasmid to visualize the transfected cells. Experiments were performed within 24–36 h after transfection.

### Immunoblotting, immunoprecipitation, surface biotinylation, and immunofluorescence

Experiments were performed as described previously^20^. Anti-BiP (ab21685), anti-β-actin (ab6276, Abcam, Cambridge, UK), anti-Myc (sc-40), anti-aldolase A1 (sc-12059, Santa Cruz Biotechnology, Dallas, TX, USA), anti-GOLGB1 (HPA011008), and anti-FLAG (F3165, Sigma–Aldrich, St. Louis, MI, USA) antibodies were purchased from commercial sources. Coimmunoprecipitation was performed using EZview Red Anti-FLAG M2 and anti-c-Myc Affinity Gel (Sigma). Surface biotinylation was performed using 0.3 mg/mL EZ-Link Sulfo-NHS-SS-Biotin and NeutrAvidin (Thermo Fisher Scientific). Immunoblotting was performed using primary antibodies at a 1:1000 dilution, followed by corresponding anti-isotype secondary antibodies (Santa Cruz Biotechnology) at a 1:2000 dilution. Signals were visualized using the SuperSignal West-Pico Kit (Thermo Fisher Scientific). For immunofluorescence, blocking buffer containing 10% donkey serum and 1% bovine serum albumin in phosphate buffered saline was used, and dilutions of primary and fluorophore-tagged secondary antibodies were 1:100 and 1:2000, respectively. Confocal images were obtained with a Carl Zeiss LSM780 instrument; ZEN software was used for image processing.

### Electrophysiology assay

Whole-cell patch clamp techniques were used for measuring channel activity in *KCNQ4*-expressing CHO-K1 cells. Cells were transferred to a bath mounted on the stage of an IX-71 inverted microscope (Olympus, Osaka, Japan) equipped with a light source set to green fluorescence excitation wavelengths. Patch clamp experiments were performed at room temperature (23 °C–25 °C). Microglass pipettes (World Precision Instruments, Sarasota, FL, USA) were fabricated using a PC-10 dual-stage glass micropipette puller (Narishige, Tokyo, Japan) and an MF-830 microforge with a resistance of 2.0–2.5 MΩ (Narishige, Tokyo, Japan). Currents were recorded using an Axopatch 200B amplifier and Digidata 1440 A interface, digitized at 10 kHz and low pass-filtered at 5 kHz by pClamp software v. 10.3 (Molecular Devices, Sunnyvale, CA, USA). The series resistance was compensated by an offset circuit in Axopatch 200B. All voltage and current trace data were analyzed using Clampfit v. 10.3 and Origin v. 8.0 software (Microcal, Northampton, MA, USA). The step pulse protocol used for KCNQ4 channel recordings started from a holding potential of −80 mV, depolarized for 2 s from −80 mV to 20 mV with 20 mV increments, and followed by a tail pulse at −50 mV for 0.5 s. Whole-cell patch clamp analysis was conducted using a bath solution containing 147 mM NaCl, 5 mM KCl, 1.5 mM CaCl_2_, 1 mM MgCl_2_, 10 mM HEPES, and 10 mM glucose (adjusted to pH 7.4 with NaOH). The pipette solution contained 130 mM KCl, 10 mM HEPES, 1 mM CaCl_2_, 10 mM EGTA, and 3 mM Mg-ATP (adjusted to pH 7.2 with KOH).

### Fluorescence-based thallium flux assay

CHO-K1 cells were transfected in 96-well plates with *KCNQ4* variants or empty plasmids. After 48 h, the medium was replaced with 80 μL/well of FluxOR (Invitrogen) loading buffer and incubated for 1 h at 37 °C in the dark. The loading buffer was removed, and 100 µL of assay buffer was added to each well. To activate the KCNQ4 channels, cells were pretreated with 10 μM retigabine for 10 min. FluxOR fluorescence (excitation/emission: 490/525 nm) was recorded 10 s prior to the addition of 20 µL of the stimulus buffer containing a low level of thallium ions, and the fluorescence was monitored for an additional 15 s. FluxOR fluorescence was recorded using a Zyla sCMOS camera (Andor Technology) and a Nikon Eclipse Ti inverted microscope (Nikon Instruments) and analyzed using Metamorph analysis software (Molecular Device). All buffers were prepared according to the manufacturer’s instructions.

### Three-dimensional structure modeling

To examine the effects of the variants at the protein level, structural modeling of KCNQ4 was performed using Swiss-Model (https://swissmodel.expasy.org/). Amino acid residues surrounding the pore region (residues 270–345) of KCNQ1 of *Xenopus laevis* (PDB 5vms), which shares 70% sequence identity with human KCNQ4 (resides 256–326), was used as the template structure. The tertiary structure of the domains was generated, and the 3D structures of the normal and mutated proteins were visualized using the UCSF Chimera package (http://www.cgl.ucsf.edu/chimera). Protein stability was predicted using Site-Directed Mutator (http://marid.bioc.cam.ac.uk/sdm2/), I-Mutant Suite (http://gpcr2.biocomp.unibo.it/cgi/predictors/I-Mutant3.0/I-Mutant3.0.cgi), and CUPSAT (http://cupsat.tu-bs.de/).

## Results

### DFNA2-associated *KCNQ4* variants

**Fig. 1:**
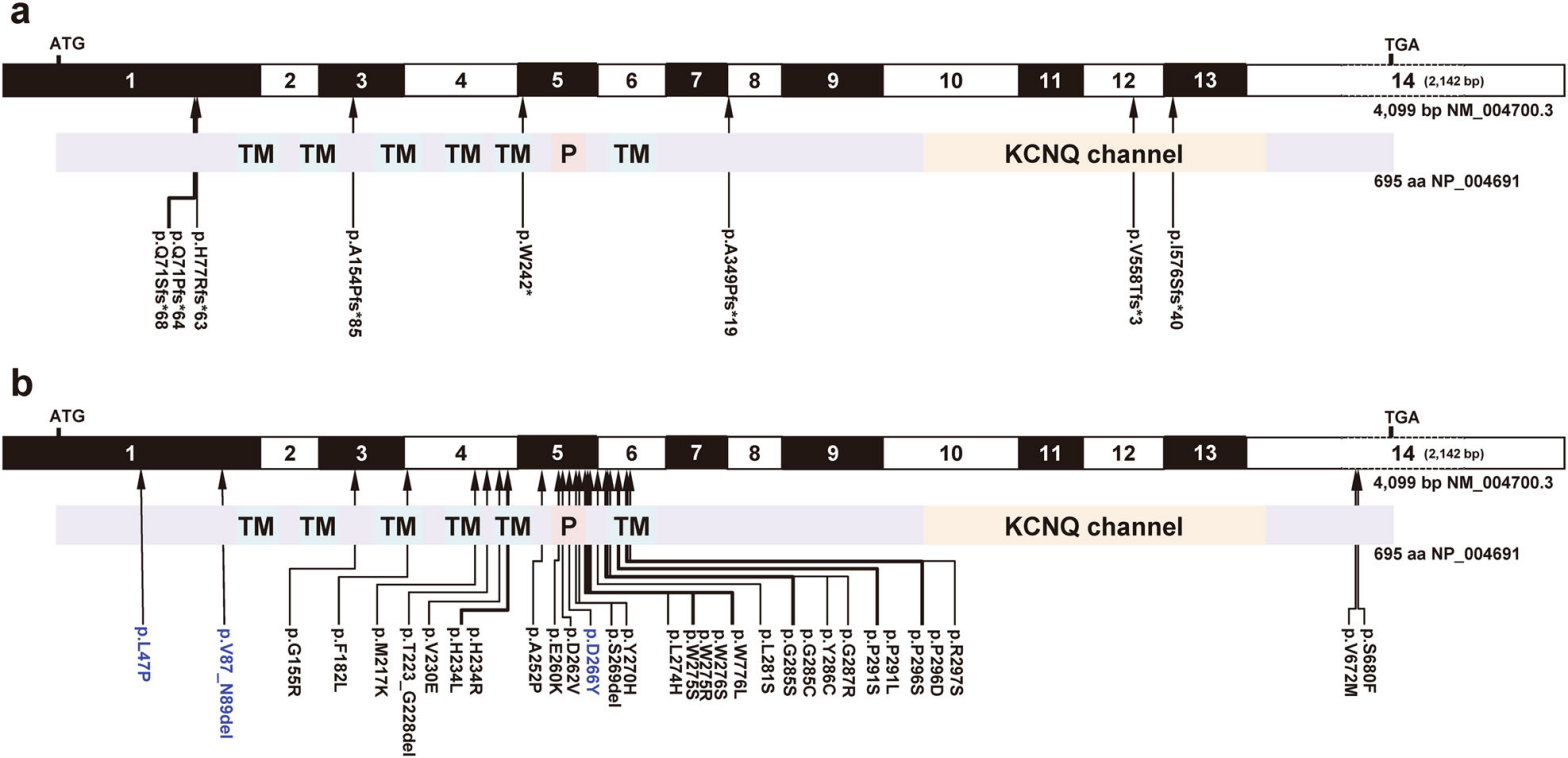
Autosomal dominant non-syndromic hearing loss (DFNA2)-associated *KCNQ4* variants. **a** Exon structure of human *KCNQ4* cDNA and domain structure of KCNQ4 are shown. Only variants identified in individuals with DFNA2 are shown. *KCNQ4* contains 14 exons; the positions of the start (ATG) and stop (TGA) codons are indicated. Loss-of-function mutations, such as nonsense and frameshift mutations, are indicated. **b** Missense and in-frame deletion mutations are indicated. Note that mutations are clustered in exons 5 and 6, which encode part of the transmembrane (TM) 5, pore region, and TM6. The pathogenic variants identified by our group are shown in blue

In HGMD Professional, 36 *KCNQ4* variants have been reported, with most of them linked to ADNSHL except the c.1525 G > T;p.E509* variant, which is associated with autism spectrum disorder (Fig. 1). We reported three pathogenic variants, including one founder mutation, in Koreans (Fig. 1b)^21,22^. Among the pathogenic variants of KCNQ4, there are also three in-frame deletions (Fig. 1b). In addition, c.648 C > T is a synonymous variant but was detected in an individual with DFNA2.

In ClinVar, 106 *KCNQ4* variants have been reported, 24 of which are also found in HGMD Professional. Of the 82 *KCNQ4* variants reported only in ClinVar, 70 are single nucleotide changes, and 12 are copy number variations (CNVs) that span large genomic regions affecting multiple genes, including *KCNQ4*. Of the 70 single nucleotide variants, five are pathogenic or likely pathogenic, 35 are benign or likely benign, and 30 are of uncertain significance. Of the 12 CNVs in ClinVar, nine are pathogenic, and three are of uncertain significance. Nine pathogenic CNVs, including *KCNQ4*, are mostly linked to severe phenotypes, such as global developmental delay, intellectual disability, and cardiac defects. One CNV, a duplication of chr1:849,467-248,224,649 (hg19), is associated with ear anomaly, short stature, cardiovascular anomaly, and polydactyly.

### *KCNQ4* variants in the general population

The gnomAD currently contains 123,136 exome sequences and 15,496 whole-genome sequences from unrelated individuals. There are 1110 *KCNQ4* variants in gnomAD, of which 297 are missense or loss-of-function (LoF) variants. Of the 19 LoF *KCNQ4* variants, 15 are nonsense or frameshift, and four affect an obligatory splice site (Table 1). The minor allele frequency (MAF) of these LoF alleles is very low (less than 0.0003234). None of them has been reported in HGMD Professional or ClinVar, and their pathogenicity or clinical significance is unknown. However, given that c.211delC;p.Q71Sfs*68 and c.725 G > A;p.W242* are reported as pathogenic in HGMD Professional, seven LoF variants (p.H77Lfs*159, p.R95P*44, p.H102 Tfs*37, p.Q122*, c.405+2T>G, p.Y160*, and p.Y232*) might be pathogenic, but this needs to be experimentally demonstrated.

**Table 1:** Loss-of-function KCNQ4 variants from the genome Aggregation Database

hg19	cDNA position	Amino acid substitution	dbSNP150	gnomAD MAF	gnomAD POPMAX	CADD
chr1:41249995- > T	c.229_230insT	p.H77Lfs*159	rs748123571 With Pathogenic allele T = 0.00001/1 (ExAC)	0.000004434 (no hom)	SAS (0.000033)	27.5
chr1:41250049 G > -	c.284delG	p.R95Pfs*44	rs773336330 - = 0.000009/1 (ExAC)	0.000004319 (no hom)	NFE (0.00001)	28.1
chr1:41250068 C > -	c.304delC	p.H102Tfs*37	No	0.000004322 (no hom)	NFE (0.00001)	No
chr1:41282986 C > T	c.364C>T	p.Q122*	No	0.0000323 (no hom)	AFR (0.000115)	36
chr1:41283029T>G	c.405+2T>G		No	0.000004087 (no hom)	NFE (0.000009)	23.5
chr1:41283910C>G	c.480C>G	p.Y160*	No	0.000004062 (no hom)	NFE (0.000009)	36
chr1:41284340C>A	c.696C>A	p.Y232*	No	0.00003234 (no hom)	AFR (0.000115)	37
chr1:41285137- > G	c.831dupG	p.T278Dfs*11	rs749884791 G = 0.000008/1 (ExAC)	0.000004061 (no hom)	NFE (0.000009)	35
chr1:41289914T>-	c.1277delT	p.F426Sfs*70	No	0.000007795 (no hom)	AFR (0.000046)	No
chr1:41292308A>G	c.817-2A>G		No	0.000008099 (no hom)	Other (0.000287)	20.8
chr1:41300687- > ACCGT	c.1664_1668dupCGTAC	p.D557Rfs*4	rs761127546	0.000004061 (no hom)	EAS (0.000058)	35
chr1:41300693C>G	c.1668C>G	p.Y556*	rs140945833	0.00001624 (no hom)	EAS (0.000116)	38
chr1:41300749 G > -	c.1725delG	p.I576Sfs*40	No	0.00006463 (no hom)	NFE (0.000133)	36
chr1:41303336 G > -	c.1747delG	p.V583Wfs*33	No	0.000004115 (no hom)	NFE (0.000009)	No
chr1:41303467 G > A	c.1875+1G>A		No	0.000004384 (no hom)	AMR (0.000031)	26.1
chr1:41303468T>A	c.1875+2T>A		rs748481952 A = 0.00001/1 (ExAC) A = 0.00007/2 (TOPMED)	0.000008776 (no hom)	NFE (0.000019)	25.6
chr1:41304098- > TC	c.1991_1992dupTC	p.T665Sfs*77	rs765842914 T = 0.000008/1 (ExAC)	0.000004065 (no hom)	NFE (0.000009)	34

*gnomAD* genome Aggregation Database, *No* no data

Of the 278 missense *KCNQ4* variants in gnomAD, we examined the changing amino acid residues between the transmembrane domains (TM) 5 and 6 (aa 259–296) surrounding the pore region (aa 271–292), as most known *KCNQ4* variants linked to hearing loss are clustered around this region^7^. There are six variants in this region of KCNQ4 in gnomAD, and none of them has been reported in either HGMD Professional or ClinVar except p.T278A, which has been reported in ClinVar as of unknown significance (Table 2 and Supplementary Fig. 1). These variants are rare, and their MAFs are less than 0.00003. In addition, their pathogenicity or clinical significance is unknown.

**Table 2:** Missense variants corresponding to the pore region of KCNQ4 from the genome Aggregation Database

hg19	cDNA position	Amino acid substitution	Conservation				dbSNP150	gnomAD MAF	gnomAD POPMAX	Mutation Taster	PP2 Humvar	SIFT	Con-del	CADD
			Mm	Gg	Xt	Dr								
chr1:41285101 G > A	c.791A>G	p.N264S	N	N	N	N	rs745846744 G = 0.00002/2 (ExAC)	0.0000121831 (no hom)	SAS (0.000097)	DC (1)	Dam (0.996)	Del (0.02)	Del (0.845)	24.2
chr1:41285116C>T	c.806C>T	p.S269F	S	T	T	T	rs771549225 T = 0.000008/1 (ExAC)	0.00000406098 (no hom)	AMR (0.000030)	DC (0.999)	Dam (0.876)	Del (0)	Del (0.786)	32
chr1:41285127T>G	c.817T>G	p.S273A	S	S	S	A	rs763733527 G = 0.00005/6 (ExAC)	0.0000284273 (no hom)	SAS (0.000227)	DC (0.999)	Dam (0.992)	Tol (1)	Neu (0.448)	12.32
chr1:41285142A>G	c.832A>G	p.T278A	T	T	T	T	rs763326539 G = 0.000008/1 (ExAC) G = 0.00003/1 (TOPMED)	0.00000406157 (no hom)	AMR (0.00003)	DC (1)	Dam (0.996)	Tol (0.33)	Del (0.486)	24.2
chr1:41285553T>A	c.841T>A	p.L281M	L	L	L	L	No	0.00000406055 (no hom)	NFE (0.000009)	DC (1)	Dam (0.999)	Del (0)	Del (0.935)	24.6
chr1:41285596T>C	c.884T>C	p.L295P	L	L	L	T	No	0.00000406296 (no hom)	AFR (0.000065)	DC (1)	Dam (0.94)	Tol (0.3)	Neu (0.414)	24.6

*Ben* benign, *DC* disease-causing, *Del* deleterious, *hom* homozygous, *gnomAD* genome Aggregation Database, *No* no data, *Neu* neutral, *PP2* PolyPhen-2 prediction score Humvar, *SIFT* Sorting Intolerant from Tolerant

We also examined a whole-genome sequencing dataset of 397 Korean individuals and found six missense *KCNQ4* variants, but none of them corresponded to the pore region of the protein (Supplementary Table 1).

### Functional effects of *KCNQ4* variants around the pore region

As the effects on KCNQ4 function of the six missense variants around the pore region reported in gnomAD have not been investigated, we aimed to characterize these variants and a missense variant (p.R433W) detected in the Korean dataset.

**Fig. 2:**
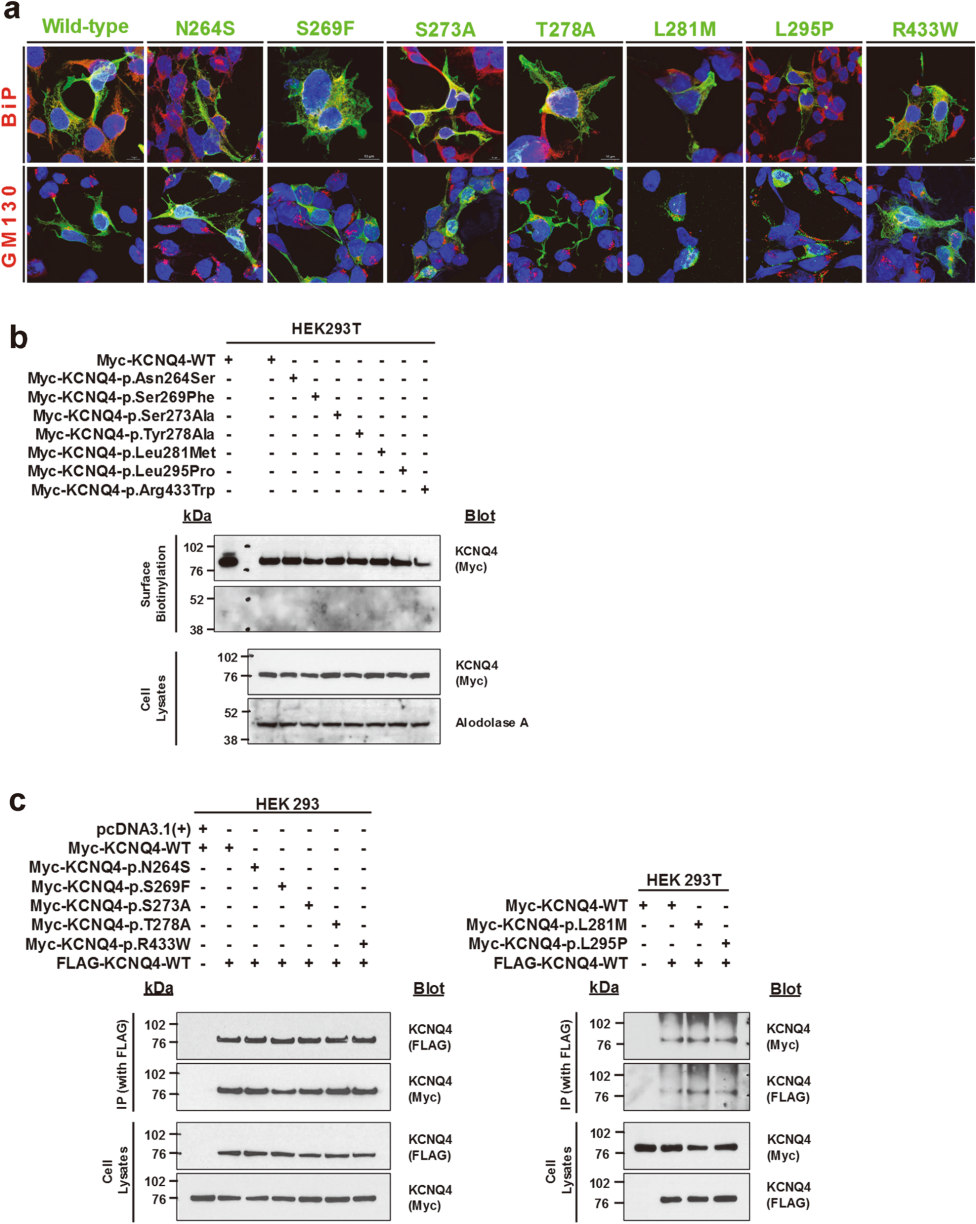
Effects of *KCNQ4* variants on protein expression on the cell surface and on subunit interaction. **a** Immunofluorescence of wild-type (WT) and variant KCNQ4 proteins in HEK 293 cells. Cells were immunostained with anti-Myc, anti-BiP, and anti-GOLGB1 antibodies. Nuclei were stained with DAPI. BiP and GOLGB1 indicate the endoplasmic reticulum and Golgi apparatus, respectively. All KCNQ4 variant proteins were observed on the plasma membrane. **b** Cell surface biotinylation. Proteins on the plasma membrane were labeled with biotin, isolated with avidin beads, and assessed by western blotting. Surface expression of KCNQ4 variant proteins was similar to that of the WT protein. **c** Coimmunoprecipitation. Cells were cotransfected with FLAG-tagged WT KCNQ4 and Myc-tagged WT or mutant KCNQ4 clones. After transfection (36 h), whole-cell lysates were subjected to immunoprecipitation using anti-FLAG beads and immunoblotted. All KCNQ4 variant proteins interacted with the WT protein

We first performed immunofluorescence and surface biotinylation assays to examine the expression levels of the WT and variant KCNQ4 proteins at the plasma membrane in HEK 293 cells. Using immunofluorescence with endoplasmic reticulum and Golgi markers, the WT and all variant KCNQ4 proteins were detected at the cell surface (Fig. 2a). Surface biotinylation assays also confirmed that all variant proteins reached the plasma membrane (Fig. 2b). The expression levels of variant proteins were comparable to that of WT KCNQ4 even when overexpressed (Fig. 2b).

Because the KCNQ4 channel functions as a homo or heterotetramer of subunits^10^, we examined whether variant KCNQ4 proteins affected heteromeric assembly with the WT protein. Coimmunoprecipitation demonstrated that variant proteins were able to interact with the WT protein, indicating that heterotetramers containing the WT and variant proteins can form (Fig. 2c). Taken together, these results demonstrate that variant proteins do not exhibit defects in trafficking or subunit assembly.

**Fig. 3:**
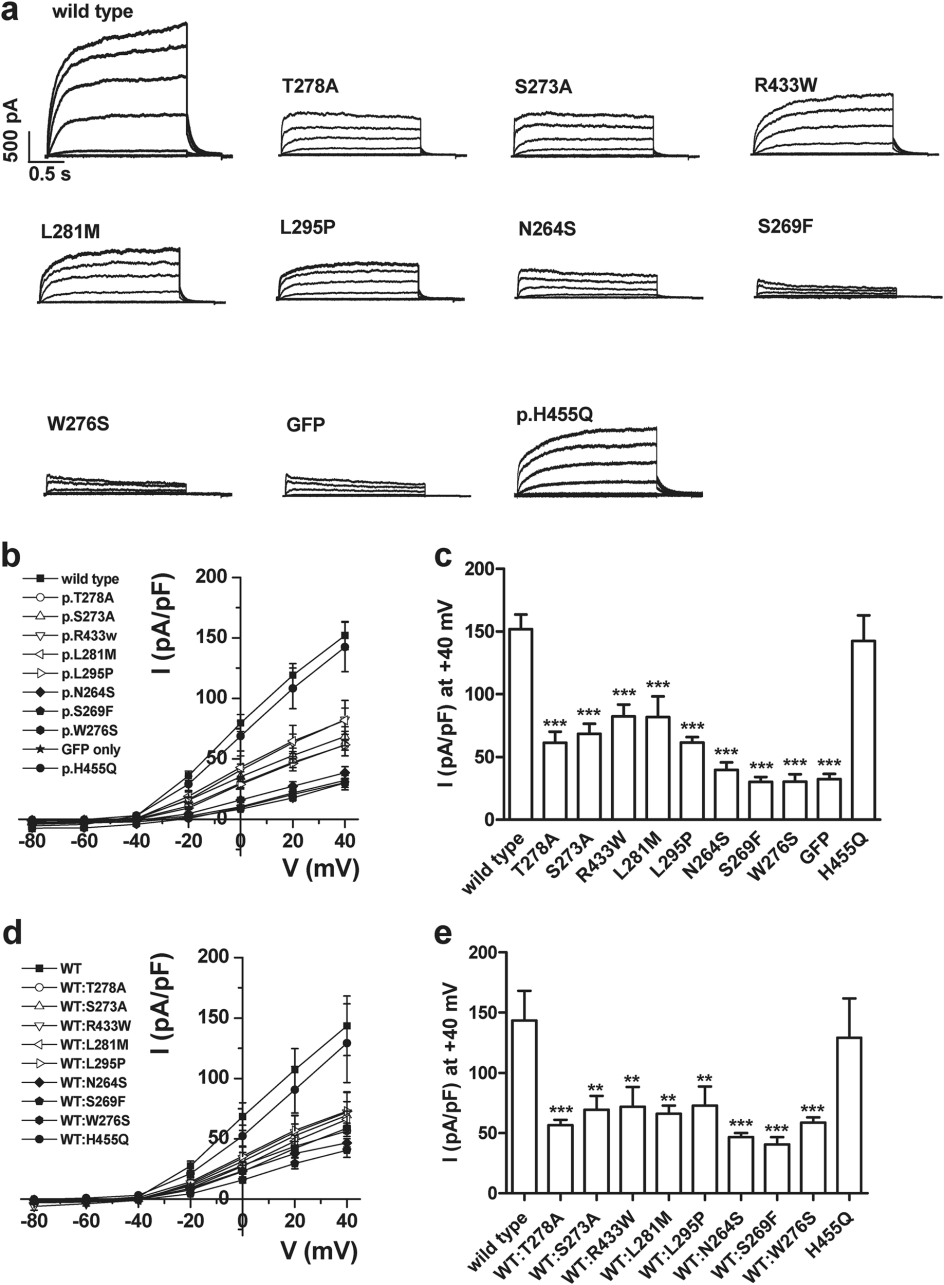
Whole-cell currents produced by KCNQ4 mutants. **a** Representative whole-cell current traces recorded in CHO cells transfected with wild-type (WT) and mutant KCNQ4 with step pulses from −80 mV to 40 mV in 20 mV steps. **b** Mean current-voltage (I–V) relation curves obtained from steady-state currents elicited at each step pulse voltage. **c** Summary bar graph of steady-state currents elicited at 40 mV normalized to membrane area (capacitance), obtained from whole-cell current recordings as presented in A. Averaged whole-cell current densities were 151.9 ± 11.6 pA/pF (*n* = 30), 61.5 ± 8.8 pA/pF (*n* = 19), 68.6 ± 7.8 pA/pF (*n* = 17), 81.8 ± 16.4 pA/pF (*n* = 12), 61.6 ± 4.3 pA/pF (*n* = 15), 82.3 ± 9.5 pA/pF (*n* = 19), 38.5 ± 5 pA/pF (*n* = 16), 30.2 ± 3.8 pA/pF (*n* = 12), 30.4 ± 5.9 pA/pF (*n* = 8), 142.5 ± 20.5 pA/pF (*n* = 4), and 32.4 ± 4.2 pA/pF (*n* = 9) for WT, p.T278A, p.S273A, p.L281M, p.L295P, p.R433W, p.N264S, p.S269F, p.W276S, p.H455Q and green fluorescent protein only, respectively. Data represent the mean ± SEM. ***P < 0.001 compared to WT. Statistical analysis was performed using one-way ANOVA with Bonferroni’s multiple comparison. **d** Dominant-negative effects of mutants on KCNQ4-mediated currents were analyzed with a 1:1 ratio of WT and mutants KCNQ4 and step pulses from −80 mV to 40 mV in 20 mV steps. The mean I–V relation curve obtained from the steady-state current values elicited at each step pulse voltage is shown. **e** Summary bar graph of the steady-state currents elicited at 40 mV normalized to capacitance. The averaged whole-cell current densities were 143.6 ± 24.5 pA/pF (*n* = 7), 56.6 ± 4.5 pA/pF (*n* = 11), 69.4 ± 1.1 pA/pF (*n* = 9), 72 ± 16.4 pA/pF (*n* = 12), 66.1 ± 6.7 pA/pF (*n* = 10), 72.9 ± 15.9 pA/pF (*n* = 10), 46.7 ± 3.4 pA/pF (*n* = 9), 40.6 ± 6.04 pA/pF (*n* = 9), 58.8 ± 4.2 pA/pF (*n* = 10), and 129.2 ± 32.5 pA/pF (*n* = 4) for WT, p.T278A, p.S273A, p.R433W p.L281M, p.L295P, p.N264S, p.S269F, p.W276S, and p.H455Q, respectively. The red line is the predicted current density if there is no dominant-negative effect. Data represent the mean ± SEM. **P < 0.01, ***P < 0.001 compared to the WT. Statistical analysis was performed using one-way ANOVA with Bonferroni’s multiple comparison

To determine the effects of KCNQ4 variants on channel function, whole-cell currents were recorded in CHO-K1 cells transfected with WT or variant *KCNQ4* clones. The p.W276S mutation, which is known to cause DFNA2^3^, was used as a positive control^23^, and a common single nucleotide polymorphism (rs34286752, c.1365T>G; p.H455Q), the MAF of which is 0.2584, was also included in the functional study. All variants showed reduced potassium channel activity (Fig. 3a, b and Supplementary Fig. 1). Pore region variants p.T278A, p.S273A, p.L281M, and p.L295P as well as p.R433W produced currents of ~40–53% of WT levels (Fig. 3c and Supplementary Fig. 2). In addition, p.N264S and p.S269F showed no channel function as they produced basal level currents similar to those recorded in GFP- or p.W276S-transfected CHO cells (Fig. 3a–c and Supplementary Fig. 2). Because DFNA2-associated *KCNQ4* variants are present in a heterozygous state and exhibit a dominant-negative effect on functional WT channels, thereby decreasing potassium currents^12,23^, we also examined whether six missense *KCNQ4* variants around the pore region reported in gnomAD had a dominant-negative effect. We measured potassium currents in CHO cells cotransfected with WT and mutants (1:1 ratio) to mimic the heterozygous condition, and the results show that all six variants exerted dominant-negative inhibitory effects on WT KCNQ4-mediated currents (Fig. 3d, e, and Supplementary Fig. 3).

### Rescue of potassium currents produced by KCNQ4 variants by retigabine

**Fig. 4:**
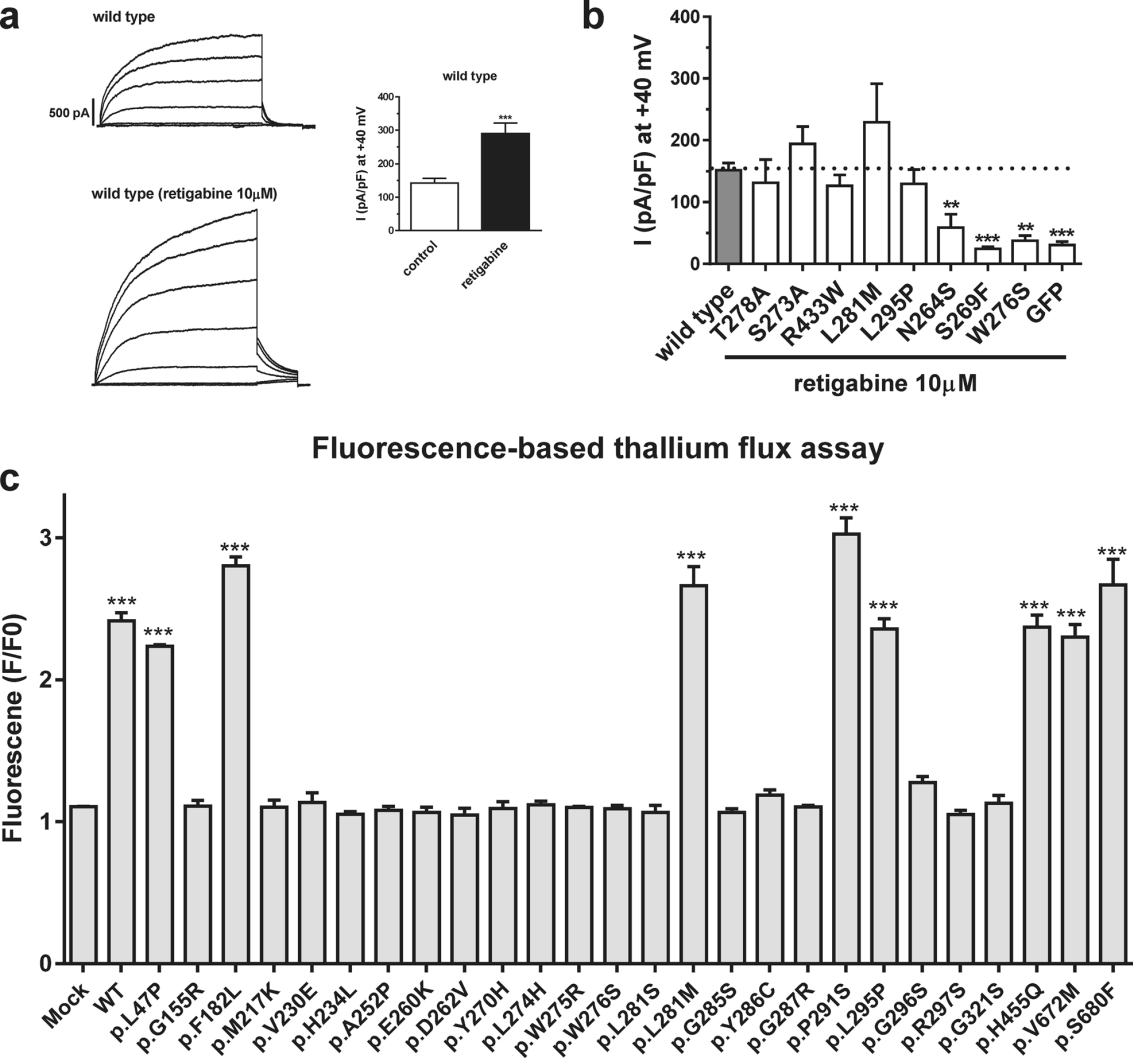
Effect of retigabine on current produced by KCNQ4 mutants. **a** Enhancement of voltage-gated potassium channel activity of wild-type (WT) KCNQ4 by retigabine. **b** Summary bar graph of current produced by KCNQ4 mutants and rescued by retigabine versus that of WT, obtained from steady-state currents elicited at 40 mV shown in Supplementary Fig. 2. Current values were 131.4 ± 37.6 pA/pF (*n* = 11), 194 ± 28.3 pA/pF (*n* = 9), 229.2 ± 62.2 pA/pF (*n* = 6), 129.7 ± 23.2 pA/pF (*n* = 8), 126.4 ± 17.8 pA/pF (*n* = 10), 58.8 ± 21.5 pA/pF (*n* = 6), 24.4 ± 3.3 pA/pF (*n* = 4), 37.4 ± 8.6 pA/pF (*n* = 4), and 30.4 ± 5.8 pA/pF (*n* = 5) for p.T278A, p.S273A, p.L281M, p.L295P, p.R433W, p.N264S, p.S269F, p.W276S, and green fluorescent protein, respectively. **c** Effect of retigabine on thallium influx in cells expressing WT and mutant KCNQ4. Data represent the mean ± SEM. **P < 0.01, ***P < 0.001 compared to WT (**a**, **b**) or mock (**c**). Statistical analysis was performed using one-way ANOVA with Bonferroni’s multiple comparison

Retigabine was developed as an anticonvulsant to treat epilepsy, acting primarily as a potassium channel (including KCNQ4) opener^24^. Retigabine increased KCNQ4-mediated whole-cell current by more than 2-fold (Fig. 4a). We examined whether the impaired conductance of KCNQ4 variants could be recovered by retigabine. As shown in Fig. 4b, the activity of p.S273A, p.T278A, p.L281M, p.L295P, and p.R433W was rescued by retigabine, whereas that of p.N264S, p.S269F, and p.W276S was not. Therefore, variants showing reduced but still-present voltage-activated currents were rescued by retigabine to almost WT levels, whereas variants with almost null potassium currents were obviously nonrescuable (Fig. 4b). We also examined the effect of retigabine on DFNA2-associated *KCNQ4* variants reported in HGMD and measured ion conductance using the thallium-sensitive fluorescent dye FluxOR. Variants p.L47P, p.F182L, p.L281M, p.P291S, p.L285P, p.V672M, and p.S680F were rescued by retigabine (Fig. 4c), indicating that only some DFNA2-associated *KCNQ4* variants are rescued by retigabine. Interestingly, even though the variants occurred at the same amino acid residue, p.L281S did not respond to retigabine, whereas p.L281M was rescued by the drug (Fig. 4c).

To examine the effects of the variants at the protein level, we performed structural modeling of WT and variant KCNQ4 proteins based on the cryo-EM structure of *Xenopus* KCNQ1^25^. The p.W276S variant was predicted to decrease the stability of KCNQ4 by three prediction programs (Supplementary Fig. 4). Six missense variants around the pore region were also predicted to have a destabilizing effect by at least one program (Fig. S4). However, the structural prediction results did not correlate with the reduction in current mediated by the variant KCNQ4 proteins, nor did they provide insight into the differences in the rescue of voltage-gated channel activity by retigabine among the KCNQ4 variants.

## Discussion

Pathogenic *KCNQ4* variants cause progressive hearing loss inherited in an autosomal dominant manner (DFNA2)^3,10^. Although slowly progressive hearing loss is not fully attributable to the degeneration of outer hair cells in the cochlea, the main pathomechanism is the impaired potassium recycling in the endolymph, which consequently changes the nature of its electrolyte milieu and debilitates the endocochlear potential^26,27^. In mice, the mechanism of hearing loss is not haploinsufficiency but a dominant-negative effect of the mutant KCNQ4 because heterozygous knockout mice do not show any hearing loss^27^. Therefore, most mutations identified in humans are thought to have a dominant-negative effect.

The majority of variants linked to DFNA2 are located around the pore region of the protein encoded by the *KCNQ4* gene, which is responsible for the ion selectivity of the channel^12,28^. KCNQ4 mediates an M-like potassium current in the outer hair cells and type I vestibular hair cells^3,29^. The M-like potassium conductance in these cells is dominant-negatively abolished when the pore mutant p.G285S was transfected into the cells^29^. These findings indicate that the main pathomechanism of DFNA2 resulting from mutations in amino acid residues around the pore region of KCNQ4 is the dominant-negative loss of potassium current in the outer hair cells. Therefore, people with *KCNQ4* variants with impaired potassium current are strong candidates for progressive hearing loss linked to DFNA2.

In this regard, we examined all missense variants changing residues around the pore region in *KCNQ4* from public databases, including gnomAD, and found six missense variants that are present in the general population at low frequency (Supplementary Fig. 1). Because the variants have not been reported as pathogenic in HGMD or ClinVar, they are generally considered variants of unknown significance. Interestingly, we found that the *KCNQ4* variants around the pore region produced a significantly impaired potassium current. Given that several mutations in *KCNQ4* give rise to proteins with impaired trafficking, the reduced current might be attributable to reduced expression levels of the channel on the cell surface^4^. However, in our results, the *KCNQ4* variants around the pore region showed normal membrane expression levels. In addition, we demonstrated that these six missense *KCNQ4* variants from public databases can exert a dominant-negative effect. This raises the possibility that individuals carrying these variants are at higher risk of developing late-onset or progressive hearing loss. In other words, the variants can be considered risk factors for late-onset or progressive hearing loss.

Based on MAFs (q) of six missense variants around the pore region of KCNQ4 reported in gnomAD, if we assume that these variants are in Hardy-Weinberg equilibrium (p^2^ + 2pq + q^2^ = 1, p + q = 1), we can calculate the frequencies of individuals who carry these variants in a homozygous or heterozygous state (2pq+q^2^). Frequencies of heterozygous or homozygous individuals are estimated to be 0.0000244, 0.0000081, 0.0000569, 0.0000081, 0.0000081, and 0.0000081 for p.N264S, p.S269F, p.S273A, p.T278A, p.L281M, and p.L295P, respectively. Their sum is 0.0001137, which is ~5.69% of the prevalence of hearing loss (1/500 = 0.002). Therefore, *KCNQ4* variants may contribute more than expected to hearing loss.

More than one hundred genes causing NSHL have been described (http://hereditaryhearingloss.org/). NSHL is very heterogeneously inherited. In particular, autosomal dominant hearing loss is mainly related to late-onset and progressive hearing loss; therefore, it is unclear whether young children or adults are affected by hearing loss. Those with mild or moderate hearing loss are likely considered healthy because high-frequency hearing loss is commonly undetected. Thus, it is plausible that sequencing data of people who have mild-to-moderate hearing loss or who are in a subclinical stage of progressive hearing loss are present in public databases. *KCNQ4* variants attributable to ARHL or NIHL but not yet reported as pathogenic may also be included in public databases.

There are numerous ion channels in the inner ear, which are important for the maintenance of ion homeostasis and hearing function. Anion channels/transporters such as *SLC26A4* and cation channels such as *KCNQ1, KCNQE1, KCNQ4, KCNJ10, TRPV5*, and *TRPV6* are included in the list of deafness-related genes^30^. The results of the present study suggest that many unveiled pathologic variants may occur in these ion channel genes. Specifically, pore missense mutations are mainly attributable to progressive hearing loss. Further analyses are needed to uncover rare variants in these channel genes that confer a high risk for late-onset or progressive hearing loss.

KCNQ channels, particularly heteromeric KCNQ2/3, play a critical role in maintaining neuronal excitability by producing outward K^+^ currents to induce membrane repolarization and hyperpolarization^31^. Reduction in KCNQ2/3 channel activity causes neuronal disorders (hyperexcitability), such as epilepsy and tinnitus^32^. There have been many trials to develop therapies for these diseases in which KCNQ channels are activated to obtain neuronal hyperexcitability. In fact, retigabine, a small molecule that activates KCNQ2-5 channels, was approved as an anti-epileptic drug by the US Food and Drug Administration^33^. Retigabine exerts its therapeutic effect by enhancing KCNQ channel activity through the shift of voltage-dependent activation to more negative voltages, resulting in a reduction in the excessive firing of neuronal cells^34^.

KCNQ4 channels expressed in the basolateral membrane of outer hair cells play a major role in maintaining the resting membrane potential. Therefore, impaired KCNQ4 channel activity caused by dysfunctional KCNQ4 mutants induces degeneration of outer hair cells by prolonged depolarization of the membrane potential, which further leads to progressive hearing loss^35^. In our studies, retigabine rescued the voltage-activated currents mediated by KCNQ4 pore mutants, suggesting that retigabine or other KCNQ activators may be useful in the therapeutics of genetic hearing loss. There is an unmet medical need for the treatment of inherited hearing loss caused by mutations in *KCNQ4*. However, more in vitro studies and clinical trials are needed to determine the therapeutic effects of KCNQ activators on hearing loss.

Pore variants of KCNQ4 are known to be unresponsive to retigabine or zinc pyrithione, which is another KCNQ activator^36^. Similarly, our results showed that the almost null potassium activity for the two mutants was not rescued by retigabine. However, it is noteworthy that four of the six pore variants of KCNQ4 with residual voltage-activated currents were activated by retigabine to almost WT levels. Retigabine increases channel function by stabilizing the open K^+^ conducting form through binding to a single conserved tryptophan residue in the S5 domain (Supplementary Fig. 1)^37,38^. Our results show that retigabine did not enhance channel activity of variant KCNQ4 proteins with completely impaired channel function, whereas residual currents mediated by other variant KCNQ4 proteins were rescuable. Given that KCNQ activators increase KCNQ4 activity through alteration of channel conductance, it is still unclear how pore mutants are responsive to retigabine^24,39^. It is possible that retigabine induces a conformational change in the pore region. Further biophysical analyses are needed to clarify this issue.

Because KCNQ4 assembles into a homotetramer, 6.25% of tetramers are formed of WT subunits only when a heterozygous *KCNQ4* variant is present, which are potential therapeutic targets of retigabine if only the WT assembly was responsive to the drug. However, more potential retigabine targets would be available if the drug could also rescue the channel activity of pore mutant KCNQ4 as well as WT proteins.

In this context, we found that it may be possible to rescue channel activity by nonselective KCNQ activators, thereby preventing hearing deterioration in patients with DFNA2. However, retigabine has many systemic side effects, such as drowsiness, vertigo, and slurred speech, and thus has not been successful in the market^32,40^. To avoid these adverse effects, retigabine could be locally administered in the inner ear. Overall, the clinical value of this drug will be scientifically evaluated after further biophysical analysis is performed, and distinct in vivo study results are available.

In conclusion, we found that there are many unreported variants around the pore regions of KCNQ4, possibly associated with DFNA2, in public databases. Given that DFNA2 is one of the most common causes of ADNSHL, genetic testing of *KCNQ4* is highly important. Furthermore, we identified several KCNQ4 pore variants potentially treatable with a small molecule, which would enable us to realize precision medicine in genetic hearing loss.

## Supplementary information

Supplementary Information.
