# Supplementary material for: Rare KCNQ4 variants found in public databases underlie impaired channel activity that may contribute to hearing impairment
Source: Exp Mol Med. 2019 Aug 21;51(8):99. doi: 10.1038/s12276-019-0300-9 (PMC6802650; doi:10.1038/s12276-019-0300-9)
Supplement: Supplementary file 1 — Supplementary Information. [file 12276_2019_300_MOESM1_ESM.pdf]

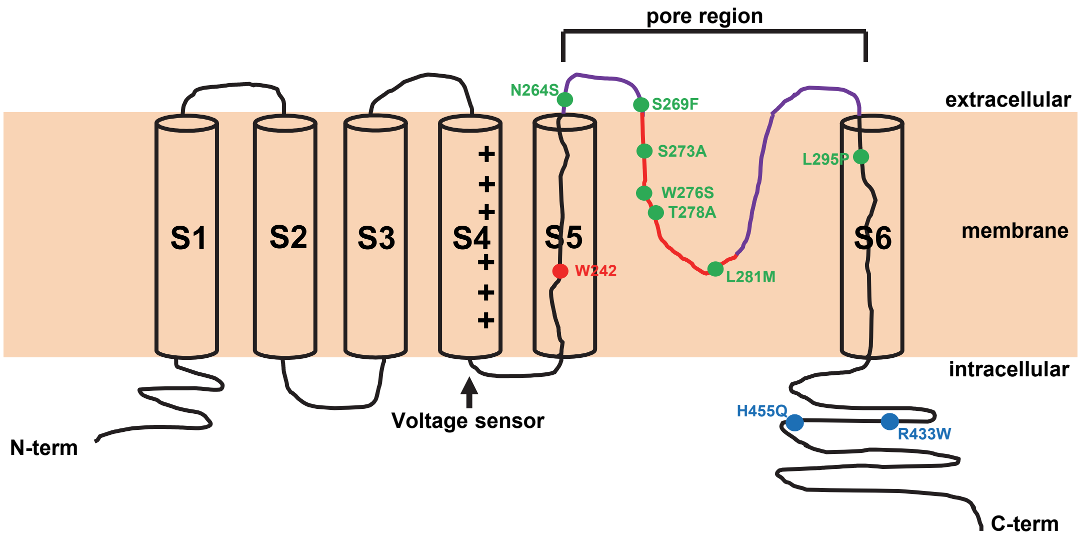

Supplementary Figure 1. Localization of KCNQ4 variants investigated in this study. The six transmembrane domains (S1-S6) and the pore region including S5, S6 domain, P-loop (purple line) and pore-helix (red line) are showed. Seven variants (green) are located in pore region and there are variants (blue) in C-terminus. Red dot indicates the conserved tryptophan (W) residue located in S5 domain. Schematic topology of human KCNQ4 is modified from Namba *et al*. (BMC Res Notes. 2012; 5: 145).

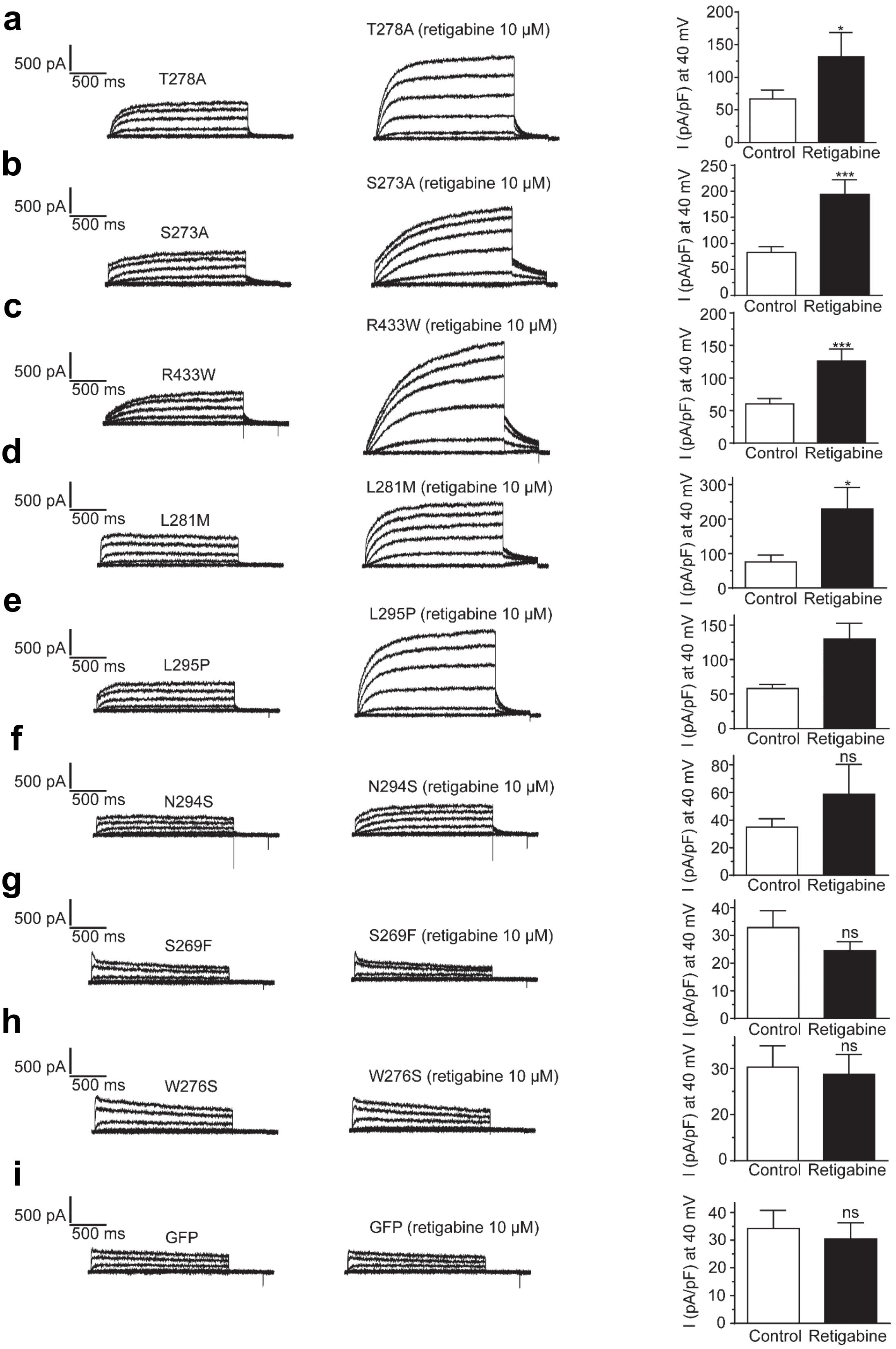

Supplementary Figure 2. a-i Representative current traces recorded in wild type and mutant KCNQ4-expressing CHO cells without (left panel) or with retigabine treatment (middle panel) A summary bar graph of retigabine-induced currents compared to the control elicited at 40 mV normalized to capacitance (right panel). Data represent the mean ± SEM. NS, no significance; * $P < 0.05$; ***$P < 0.001$ compared to control. Statistical analysis was performed using two-tailed paired t-test.

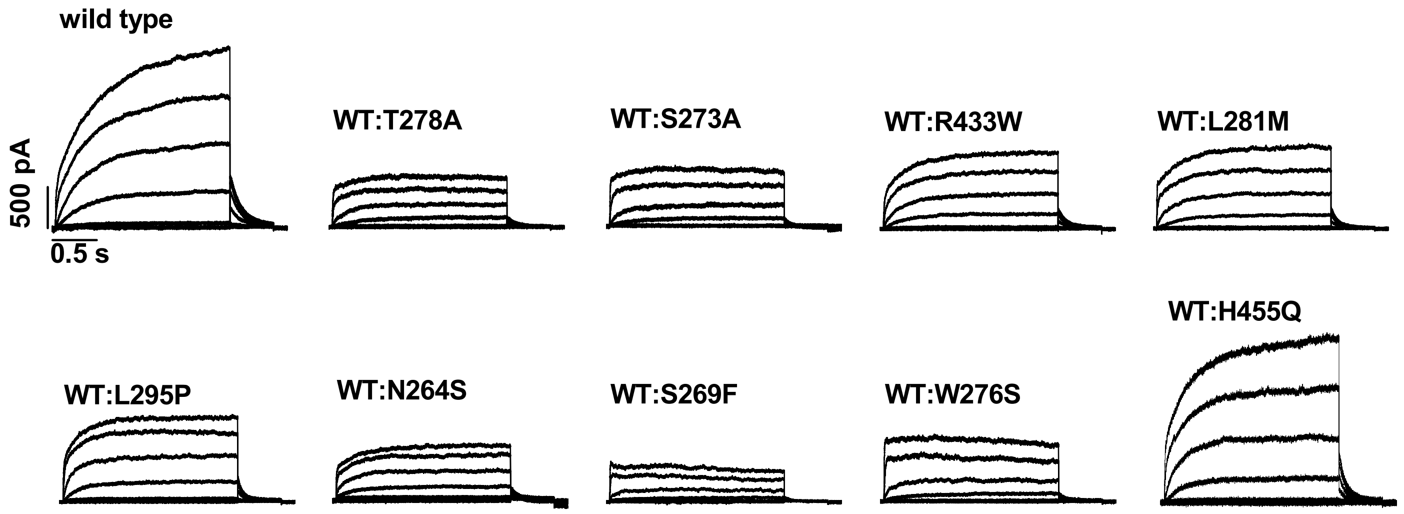

Supplementary Figure 3. Dominant-negative effects of mutants on KCNQ4 currents. Representative whole-cell current traces recorded in CHO cells transfected with 1:1 ratio of wild type and mutant KCNQ4 and with step pulses from -80 mV to 40 mV in 20 mV steps.

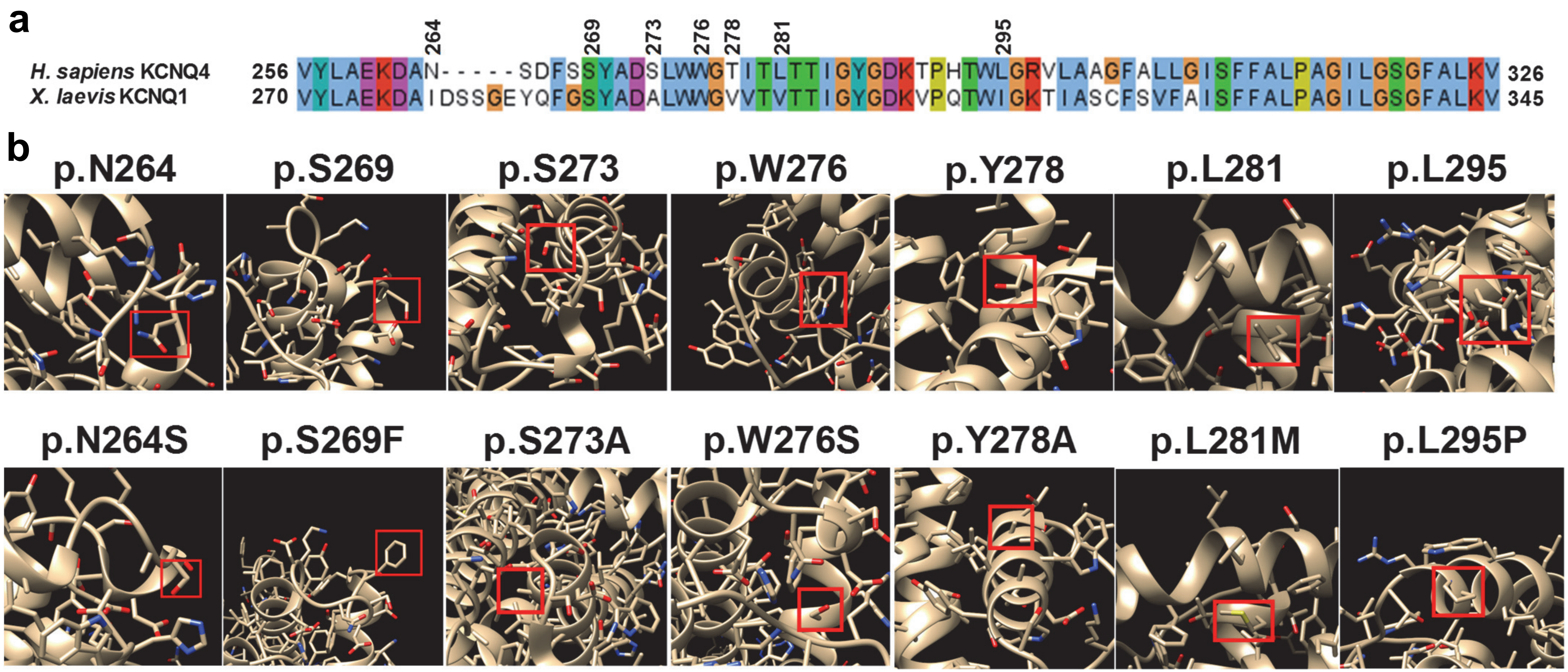

c

Amino acid change	Site Directed Mutator	I-Mutant Suite	CUPSAT
p.N264S	-1.01 (Reduced stability)	-0.54 (Decrease)	-0.416 (Reduced stability)
p.S269F	+0.04 (Increased stability)	+0.09 (Increase)	-0.03 (Reduced stability)
p.S273A	+2.53 (Increased stability)	-1.05 (Decrease)	+1.22 (Increased stability)
p.W276S	-1.95 (Reduced stability)	-1.50 (Decrease)	-0.61 (Reduced stability)
p.Y278A	1.96 (Reduced stability)	-1.14 (Decrease)	-1.23 (Reduced stability)
p.L281M	-0.68 (Reduced stability)	-0.79 (Decrease)	+0.35 (Increased stability)
p.L295P	-2.23 (Reduced stability)	-1.63 (Decrease)	-0.96 (Reduced stability)

Supplementary Figure 4. Tertiary structure prediction of KCNQ4 wild-type, p.W726S, and six missense variants around pore regions. A Amino acid sequence alignment of the pore regions of *H. sapiens* KCNQ4 and *X. laevis* KCNQ1. **b** Upper row shows wild-type residues (red box), whereas lower row show mutated residues (red box). **c** Predicted changes in protein stability by KCNQ4 variants. Numbers indicate change in Gibbs free energy (ΔΔG). Prediction follows two stage classification: ΔΔG (kcal/mol) <0 meaning the decline in protein stability and ΔΔG (kcal/mol) >0 meaning the increase in the stability of protein.

Supplementary Table 1. Missense variants of KCNQ4 detected in whole-genome sequencing data of 396 Koreans

hg19	cDNA position	Amino acid substitution	Conservation				dbSNP150	gnomAD MAF	gnomAD POPMAX	Korean WGS	Mutation Taster	PP2 Humvar	SIFT	Con-del	CADD
			Mm	Gg	Xt	Dr									
chr1:41289803 G>A	c.1165G>A	p.A389T	A	A	T	=	ND	ND	ND	0.00125945	DC (1)	Ben(0.007)	Tol(0.23)	Neu (0.042)	17.48
chr1:41296760 C>T	c.1297 C>T	p.R433W	R	K	K	K	rs760023398 T=0.00007/2 (ExAC)	ND	ND	0.00125945	DC (0.815)	Del (0.995)	Dam (0.00)	Del (0.548)	35
chr1:41296828 T>G	c.1365T>G	p.H455Q	H	H	Y	=	rs1166749791 A=0.000008/1 (TOPMED)	0.1762	FIN (0.2563)	0.0428212	PD (0.764)	Ben (0.001)	Tol (0.536)	Neu (0.021)	12.73
chr1:41296851 C>T	c.1388C>T	p.T463I	T	R	A	R	ND	ND	ND	0.00125945	Neu (0.539)	Ben (0.003)	Tol (0.17)	Ben (0.212)	22.8
chr1:41296884 C>T	c.1421C>T	p.T474I	T	P	P	S	rs758221510 T=0.00006/8 (TOPMED)	0.00006811 (no hom)	EAS (0.0009787)	0.00125945	Neu (0.999)	Ben (0.019)	Tol (0.17)	Neu (0.06)	4.89
chr1:41303409 C>G	c.1818C>G	p.D606E	D	D	E	A	rs139835231 G=0.0021/259 (TOPMED)	0.002524	EAS (0.03552)	0.0100756	Neu (0.999)	Ben (0.004)	Tol (1)	Neu (0.002)	5.36

Abbreviations: Ben, benign; DC, disease-causing; Del, deleterious; gnomAD, genome Aggregation Database; No, no data; Neu, neutral; PP2, PolyPhen-2 prediction score Humvar; SIFT, Sorting Intolerant from Tolerant.
